# Size-dependent aggregation of erythrocytes by low molecular weight hyaluronic acids of different sizes: bioactivity and quality control potential

**DOI:** 10.3389/fphys.2025.1527354

**Published:** 2025-04-01

**Authors:** Xinyue Ma, Xiao Wang, XiaoXiao Jia, Jessica H. Hui, Joshua H. Shofaro, Ran Tao, Mizhou Matthew Hui

**Affiliations:** ^1^ Biomedical Engineering, Brown University, Providence, RI, United States; ^2^ Department of Hepatobiliary-Pancreatic Surgery, Zhejiang Provincial People’s Hospital, Hangzhou, China; ^3^ College of Animal Science and Technology, Qingdao Agriculture University, Qingdao, China; ^4^ Stanford Cancer Institute, School of Medicine, Stanford University, Stanford, CA, United States; ^5^ College of Letters and Science, University of California, Santa Barbara, Santa Barbara, CA, United States

**Keywords:** hyaluronan (HA), low molecular weight HA (LMW-HA), red blood cell aggregation, biological activity, binding capacity

## Abstract

**Introduction:**

Hyaluronic acid (HA) is a crucial biological molecule whose diverse functions are strongly influenced by its molecular weight. In particular, low molecular weight HA (LMW-HA) fragments—such as HA60 (average 60 kDa), HA35 (average 35 kDa), and HA24 (average 24 kDa)—exhibit enhanced tissue permeability and unique interactions with cell surfaces compared to high molecular weight HA (HMW-HA). This study investigates the size-dependent aggregation effects of LMW-HA on erythrocytes and examines the implications for bioactivity, quality control, and therapeutic applications.

**Methods:**

We investigated the effects of LMW-HA fragments on erythrocyte aggregation across molecular sizes using erythrocyte sedimentation rate (ESR) assays, CD44 receptor blocking assays, and molecular weight assessment via gel electrophoresis and GPC-MALLS. LMW-HA samples were applied at varying concentrations to measure their binding affinity to erythrocytes, while CD44 antibodies were used to assess receptor involvement. Species-specificity of aggregation was examined by comparing erythrocytes from different animals.

**Results:**

LMW-HA induced erythrocyte aggregation in a size-dependent manner, with HA60 exhibiting the strongest binding affinity, followed by HA35 and HA24. Aggregation was partially reversible and could be inhibited by CD44 antibodies, indicating a receptor-mediated interaction. Minimum effective concentrations for aggregation were inversely related to molecular weight, with lower molecular weight fragments requiring higher concentrations. Species-specific effects were also observed, highlighting variations in erythrocyte-HA interactions across different animals.

**Discussion:**

The study suggests that LMW-HA facilitates erythrocyte aggregation through CD44-mediated binding, offering insights into HA’s role in erythrocyte physiology and its effects on blood rheology. The findings support the potential of LMW-HA for therapeutic applications in pain and inflammation management, given its enhanced tissue permeability and reversible interaction with erythrocytes. Additionally, the size-dependent aggregation provides a valuable parameter for quality control, enabling consistency in LMW-HA products. These results underscore the importance of molecular weight in determining HA’s physiological and pharmacological activity, paving the way for further clinical research to confirm species-specific effects and optimize safe therapeutic uses of LMW-HA.

## 1 Introduction

HA is a viscous polysaccharide with high water retention and rheological properties (Li et al., 2025). It is composed of repeating disaccharide units of N-acetylglucosamine and glucuronic acid, forming HA of different molecular weights (Li et al., 2025; Jia et al., 2023). The physical properties of HMW-HA make it widely used in cosmetic fillers and knee joint injections (Iaconisi et al., 2023). With the advancement of research into the biological activities of HA, the scientific community has gradually recognized its deeper value. The naked mole rat has a HA content of up to 6% (Kulaberoglu et al., 2019), which contributes to several remarkable physiological traits, including an exceptionally long lifespan of over 30 years (Ruby et al., 2018), cancer resistance throughout its life, pain insensitivity, and low susceptibility to inflammatory diseases (Smith et al., 2011; Seluanov et al., 2018; Tian et al., 2013; Takasugi et al., 2020; Cowman et al., 2015; Kobayashi et al., 2020). These unique characteristics are thought to be closely linked to the high expression of HA synthase 2 (Has2) in the species. Research from the University of Rochester (Zhang et al., 2023) demonstrated that introducing the naked mole rat’s Has2 gene into regular mice significantly extended their lifespan and enhanced their resistance to cancer and inflammatory diseases, suggesting that Has2 may function as a longevity gene. Has2 is primarily responsible for synthesizing HMW-HA (Tian et al., 2013), which can be broken down by hyaluronidase into LMW-HA and its fragments, resulting in a wide range of HA molecular weights in the body, from oligomers to polymers in the millions of Daltons (Zhang et al., 2023).

Studies have shown that HA’s biological function is closely tied to its molecular weight and tissue permeability (Garantziotis and Savani, 2019; Cyphert et al., 2015; Marcellin et al., 2014; Ghosh et al., 2014; Campo et al., 2012). Our clinical research found that a highly purified, endotoxin-free 35 kDa LMW-HA fragment (HA35) derived from human colostrum (Jia et al., 2023; Gantumur et al., 2024) exhibits significant analgesic effects on inflammatory and neuropathic pain (Xu et al., 2024), while also effectively reducing wound swelling (Treger et al., 2024). Further studies revealed that HA35 produces analgesic effects similar to those of HMW-HA by inhibiting the activation of the capsaicin receptor TRPV1 channel (Caires et al., 2015; de la Peña et al., 2016), thereby reducing calcium ion (Ca2+) influx and alleviating skin inflammation (Shen et al., 2015). Given these findings, HA35, with its high purity and endotoxinfree nature, may offer similar biological activity to HMW-HA but with even greater therapeutic potential.

Additionally, research indicates that HA’s biological activity is linked to its interactions and binding forces between cells (Lesley et al., 2004; Zheng et al., 2023; Dovedytis et al., 2020). HA facilitates cell adhesion and movement by activating CD44 receptors on the cell surface (Johnson et al., 2018), accelerating the migration of cells, including endothelial cells, to wound sites (Solis et al., 2012). Moreover, HA interacts with various receptors, such as CD44, LYVE-1, and TLR2, to regulate the migration and secretion of immune cells, including neutrophils, macrophages, and microglial cells (Zheng et al., 2023). It also aids in the removal of lymphocytes and facilitates lymphatic fluid recirculation by binding to lymphocytes and lymphatic endothelial cells, thus reducing inflammation-induced swelling (Zhang et al., 2022). These studies suggest that HA’s biological activity is deeply connected to its ability to bind and interact with cell surfaces.

In this study, we unexpectedly discovered that specific molecular weights of LMW-HA can induce red blood cell aggregation, with the minimum concentration required for this effect varying by molecular weight. Red blood cells, despite lacking a nucleus, express high levels of CD44 on their surface, allowing them to play a role in immune responses and antigen recognition (Klei et al., 2020; Flormann et al., 2017; Lazari et al., 2020). Red blood cell aggregation is a critical factor in circulation and can affect blood flow and inflammatory responses. While Red blood cells are underexplored as a model for cell surface interaction studies (Flormann et al., 2017; Gillespie and Doctor, 2021; Barshtein et al., 2022). This research advances our understanding of how HA molecular weight influences erythrocyte aggregation and CD44-mediated interactions, offering insights into HA’s role in erythrocyte physiology and its potential therapeutic applications in inflammatory and vascular conditions.

## 2 Materials and methods

### 2.1 Experimental samples

Forearm venous blood was obtained from 10 healthy volunteers at 24 ± 4 years and collected using a 2 mL ESR tube (Kang Weishi, China) with a blood-to-sodium citrate ratio of 4:1. The protocol was formally reviewed and approved by the Ethics Committee of Qingdao Agriculture University (approval no. 2020-008), and written informed consent for the collection of human blood samples was obtained from all 10 volunteers in accordance with the Declaration of Helsinki.

Adult male beagles weighing 7 ± 2 kg (Qingdao Bolong Experimental Animal Co., Ltd., China) and twelve-week-old male BALB/c mice (Beijing Viton Lever Experimental Animal Technology Co., Ltd., China) were housed in a filter roof cage (22°C–24°C) in an animal hospital and given free access to food and water. The protocol was approved by the Qingdao Agricultural University Animal Resources Committee (QAUAR) and complies with FDA and NIH animal care guidelines (approval no. 2020-008). The beagles (n = 10), BALB/c mice (n = 30), bovine and sheep (n = 6) were fed and watered normally before the experiment, and blood was collected from the forelimb vein in the case of the beagles and tail vein in the case of the BALB/c mice, and jugular vein in the case of the bovine and sheep. A uniform collection method was used for all venous blood samples, utilizing a 2 mL ESR tube (Kang Weishi, China) with a blood-to-sodium citrate ratio of 4:1.

### 2.2 Preparation and detection of HA and HA fragments of different molecular weights

#### 2.2.1 Sources of HA with different molecular weights

HA60, HA300, and HA1600 were all purchased from Bloomage Biotech and obtained through microbial fermentation technology, with molecular weight ranges of 20–80 kDa, 300–400 kDa, and 1,000–1700 kDa, respectively. HA60 and HA300 are cosmetic grade, while HA1600 is injection grade, and all have a factory-tested purity of ≥95%. The LMW-HA HA24 (molecular weight range 20–40 kDa, purity 95%) and HA35 (molecular weight range 10–70 kDa, purity 95%) were obtained from Shaoxing Huihui Technology Co., Ltd. And prepared as follows. Additionally, the molecular weight ranges of all five HA samples used in this study were verified using agarose gel electrophoresis.

Five hundred milligrams of HA with a molecular weight of 1,600 kDa and 25 mL of ultrapure water were added to two sterile centrifuge tubes at a concentration of 20 mg/mL and repeatedly shaken and mixed until all the HA had dissolved. Then, 3 M MgCl2 and 5 M NaCl were added to final concentrations of 1 mM and 85 mM, respectively, to adjust the osmotic pressure. After repeated stirring, LHyal (Jiangnan University, provided by Prof. Jin Jian and Kang Zhen) with an initial activity of 4,300,000 U/mL was added to a final concentration corresponding to an activity of 2,500,000 U/g. rHuPH20 or SPAM1 (Shaoxing Huihui Technology Co., Ltd., China) with an initial activity unit of 27,000 U/mL was then added to a final activity of 20,000 U/g. The centrifuge tubes were placed in a shaker at 37°C for 6 h, followed by incubation in a water bath at 85°C for 45 min to inactivate rHuPH20. The tubes were filtered through a 220 nm membrane (Millipore, United States) and stored in a −20°C freezer.

#### 2.2.2 Agarose gel electrophoresis

A 1% agarose gel and 1× TBE (Solarbio, China) were used to dissolve the proteins. All samples were diluted to a final concentration of 5 mg/mL. The diluted samples were mixed 4:1 with a loading buffer. Electrophoresis was conducted at 80 V for 1 h until the sample band was visible, and the 1% agarose gel was photographed in a bright location (Jia et al., 2023; Gantumur et al., 2024).

#### 2.2.3 Gel permeation chromatography - Multi angle laser light scattering (GPC-MALLS)

Online GPC-MALLS and a refractive index detector (RID) were used to analyze the molecular weights of the samples. (1) The following chromatographic equipment and conditions were used: a GPC unit infusion pump (Waters model 515, United States), an eighteenangle laser light scatterer (Wyatt Dawn Heleos II, United States), a differential refractive index detector (Optilab Trex, United States). The chromatographic column ShodexOHpak SB-806HQ tandem SB-804HQ (8.0 mm × 300 mm, 13 μm), a mobile phase consisting of ultrapure water (0.02% sodium azide), 220 nm membrane filtration, a pH of 6, a flow rate of 1.0 mL/min, a column temperature of 40°C, an injection volume of 100 μL, and a MW measurement range of 200–109 Da (2) To determine the relative molecular mass of each sample, an appropriate amount of sample was weighed to prepare a 1 mg/mL polysaccharide solution, which was filtered for HPLC analysis. (3) To determine the molecular mass distribution, the mass concentration and light scattering intensity at different angles were measured by RID and a laser detector, respectively. The refractive index increment (dn/dc) value was 0.138. The molecular mass distribution maps of the test samples were obtained using the data processing software ASTRA (Jia et al., 2023; Gantumur et al., 2024).

### 2.3 Red blood cell agglutination assay

After cell aggregates are formed, cells exist in one of the four following states: individual cells, small cell aggregates containing 8–10 individual cells, large cell aggregates containing at least ten individual cells, or large clumps of severely deformed cells. One to 2 mL of fresh human, beagle, and BALB/c mouse venous blood was collected and mixed with HA24, HA35, HA60, HA300, and HA1600 to final concentrations of 12.00, 6.00, 3.00, 1.50, 0.75, 0.375, and 0.1875 mg/mL, respectively. Erythrocyte smears were prepared, and the saline group was used as a control. The effects of HA24, HA35, HA60, HA300, and HA1600 on the erythrocytes in erythrocyte smears were observed against a black background using a 2016 model microscope (Harbin Kangbang Black Background Optical Instrument Co., Ltd., China) equipped with a 10× eyepiece and a ×4 objective lens. Each experimental condition was repeated six times, with cells counted in each experiment, and the entire experiment was repeated six times to reduce random errors.

### 2.4 Antibody blockade studies

HA24, HA35, and HA60 were mixed with fresh human venous blood to a final concentration of 1.5 mg/mL to prepare erythrocyte smears. Observation and verification of HA35-induced erythrocyte agglutination were carried out by placing cell smears under a black background and viewing the samples by microscopy. Anti-human CD44 antibody (Abcam, United Kingdom) and rabbit IgG polyclonal isotype control (Abcam, United Kingdom) were then mixed with fresh human venous blood to a final concentration of 10 μg/mL. The mixture was incubated at 37°C for 25 min, and HA24, HA35, and HA60 were then added to final concentrations of 6.00 mg/mL, 1.50 mg/mL, and 0.375 mg/mL, respectively. Erythrocyte stained slices were prepared, and the degree to which erythrocyte agglutination was blocked by anti-human CD44 antibody was examined against a black background using a 2016 model microscope (Harbin Kangbang Black Background Optical Instrument Co., Ltd., China) equipped with a 10× eyepiece and a ×4 objective lens.

The degree of erythrocyte agglutination blockade was calculated with the following formulas to calculate the percentage of cells present as individual cells (ic%), the percentage of cells present as small cell clumps (sa%), and the percentage of cells present as large cell clumps (la%) (Xu et al., 2022).

$$ic\%=\frac{\sum_{a=1}ab}{\sum_{a=1}^{c}ab}\times 100\%$$

$$sa\%=\frac{\sum_{a=8}^{4}ab}{\sum_{a=1}^{c}ab}\times 100\%$$

$$la\%=\frac{\sum_{a=10}^{c}ab}{\sum_{a=1}^{c}ab}\times 100\%$$

where a denotes the number of cells present as a single cell or in a cell aggregate, b denotes the number of small cell aggregates, and c denotes the number of large cell aggregates. Under each condition, the experiment was repeated three times, and the average and standard deviation were calculated, with a higher standard deviation demonstrating a wider range out of the mean value and a smaller standard deviation demonstrating experimental values close to the mean value.

### 2.5 Study of the sedimentation rate and molecular weight of canine erythrocytes

In this study, ESR analysis was primarily conducted on canine blood samples due to the relative stability of canine erythrocytes in sedimentation assays and their frequent use in pharmacological studies (Diamanti et al., 2025; Hasiwa et al., 2011). While HA-induced erythrocyte aggregation was evaluated across multiple species, ESR was not measured in all species, as interspecies differences in blood rheology could introduce variability unrelated to HA-induced aggregation.

To assess the relationship between molecular weight and sedimentation rate, HA24, HA35, and HA60 were added to freshly collected venous blood from beagles at final concentrations of 3.00, 1.50, and 0.75 mg/mL, respectively. A 400 μL aliquot of each mixture was then transferred into a Westergren tube and left undisturbed for 25 min (Kratz et al., 2017). The sedimentation distance of erythrocytes was recorded, and the ESR was calculated. To minimize random errors, each experimental condition was repeated five times, and the results were averaged.

Furthermore, to validate the molecular weight and sedimentation rate relationship, six batches of HA35 for injection were mixed with freshly collected beagle venous blood at the target HA35 concentration. The mixed blood (400 μL) was then aspirated into a blood sedimentation tube and allowed to rest for 25 min. The resulting sedimentation was measured to calculate the ESR, ensuring consistency in the molecular weight and sedimentation rate range across different batches.

### 2.6 Statistical analysis

The data are expressed as the mean ± SD, and statistical analysis was performed using GraphPad Prism 6.0. For comparing the means among multiple groups, a one - way analysis of variance (ANOVA) was conducted. When the ANOVA result was significant, Tukey’s post-hoc test was used to determine which specific groups differed from each other. P > 0.05 (ns) indicated that a difference was not statistically significant. Differences for which p < 0.05 were considered statistically significant, those for which p < 0.01 were considered highly statistically significant, and p < 0.001 were considered extremely statistically significant.

## 3 Manuscript formatting

### 3.1 Molecular weights of HA and HA fragments with different sizes

#### 3.1.1 Agarose gel electrophoresis

**FIGURE 1:**
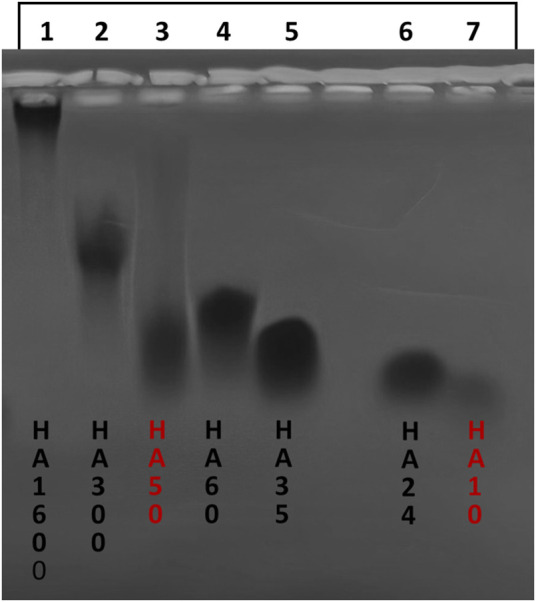
Comparison of the molecular weights of hyaluronan species and hyaluronan fragments by electrophoresis. Note: From left to right, HA1600 (lane 11,600 kDa molecular weights HA), HA300 (lane 2, 300 kDa molecular weights HA), HA50 (lane 3, 50 kDa molecular weights HA standard, Sigma), HA60 (lane 4, 60 kDa molecular weights HA), HA35 (lane 5, 35 kDa molecular weights HA), HA24 (lane 6, 24 kDa molecular weights HA), and HA10 (lane 7, 10 kDa molecular weights HA standard, Creative PEG Works).

Figure 1 shows the electrophoretic analysis of hyaluronan samples arranged from left to right as follows: HA1600 (lane 1), HA300 (lane 2), HA50 (lane 3, a 50 kDa standard with a molecular weight range of 20–80 kDa, purity 95%), HA60 (lane 4), HA35 (lane 5), HA24 (lane 6), and HA10 (lane 7, a 10 kDa standard, purity 95%). The molecular weight range for each sample is inferred from its migration relative to the standards. The bands for HA24 (lane 6) and HA35 (lane 5) are short and concentrated, indicating that these LMW-HA fragments—manufactured using LHyal and rhuPH20—have a narrow molecular weight distribution and high purity. In contrast, the extended band for HA60 (lane 4) suggests that the physical and chemical cutting method produced a broad molecular weight distribution, rendering the product less optimal. Additionally, the high molecular weight of HA1600 (lane 1) hindered its migration through the gel, and HA300 (lane 2) also exhibited limited mobility due to its relatively large size. Notably, comparison of HA24 and HA35 reveals that HA35, produced by rhuPH20 cleavage of the 1,600 kDa HA, has a larger molecular weight than HA24, which was generated by LHyal cleavage. These findings confirm that the molecular weights of LMW-HA fragments vary depending on the hyaluronidase employed, consistent with previous findings (Jia et al., 2023).

#### 3.1.2 Multi-angle light scatter gel permeation chromatography (MALLS-GPC)

We determined the molecular weights of HA24, HA35, HA60, HA300, and HA1600 by the GPC three times. As shown in Table 1, the average molecular weight of HA24 was 20.9 kDa, with a coefficient of variation of 5.53%. The average molecular weight of HA35 was 32.3 kDa, with a coefficient of variation of 4.41%. The average molecular weight of HA60 was 62.9 kDa, with a coefficient of variation of 6.24%. The average molecular weight of HA300 was 356.9 kDa, with a coefficient of variation of 5.92%. The average molecular weight of HA1600 was 1,527.5 kDa, with a coefficient of variation of 8.27%. The average molecular weight of HA1600 was 1,527.5 kDa, with a coefficient of variation of 8.27%. The experimentally determined molecular weights were consistent with the visible effects observed by electrophoresis, and the coefficients of variation were less than 10%.

**TABLE 1:** Molecular weights of five hyaluronan species determined by the GPC-MALLS method.

Hyaluronan	Average Mw/kDa	Coefficient of variation
HA24	20.9	5.53%
HA35	32.3	4.41%
HA60	62.9	6.24%
HA300	356.9	5.92%
HA1600	1,527.5	8.27%

### 3.2 Relationship between the molecular weight of HA fragments and the induction of erythrocyte agglutination in humans, beagles, and BALB/c rats

**FIGURE 2:**
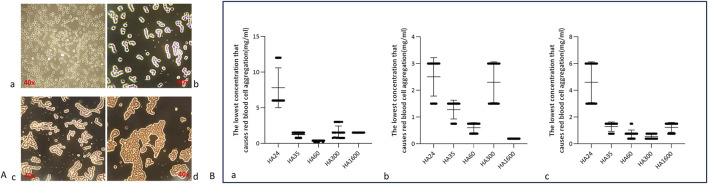
**(A)** Aggregation of human erythrocytes induced by different concentrations of hyaluronan. Note: The magnification is ×40. **(a)** The red blood cells from fresh blood in a normal state present as individual cells. **(b)** Erythrocytes contained in small agglomerates of 8–10 individual cells in a “curved coin roll” state. **(c)** Erythrocytes contained in large clumps of at least 10 single cells in a “curved coin roll” agglutination state. **(d)** The state of the cell when it is severely deformed. **(B)** Scatterplots of five hyaluronan species concentrations that induced the agglutination of erythrocytes from different animals. Note: These data represent five independent experiments, each conducted with six replicates. Specifically. **(a)** Minimum concentrations of HA24, HA35, HA60, HA300, HA1600 that induced the agglutination of human erythrocytes (n = 6). **(b)** Minimum concentrations of HA24, HA35, HA60, HA300, and HA1600 that induced the agglutination of beagle erythrocytes (n = 6). **(c)** Minimum concentrations of HA24, HA35, HA60, HA300, and HA1600 that induced the agglutination of BALB/c mouse erythrocytes (n = 6).

The state of red blood cells in normal blood is shown in Figure 2A: the cells exist independently and do not form aggregates. In this study, HA was found to induce the agglutination of red blood cells (namely, erythrocyte rouleaux formation) in animal blood. Hyaluronan of different molecular weights induced erythrocyte rouleaux formation at certain concentrations (Figures 2Ac, d). HMW-HA caused severe erythrocyte rouleaux formation (Figures 2Ac, d). This suggests that LMW-HA fragments of different molecular weights induced erythrocyte rouleaux formation by binding to or acting at the surface of red blood cells.

**FIGURE 3:**
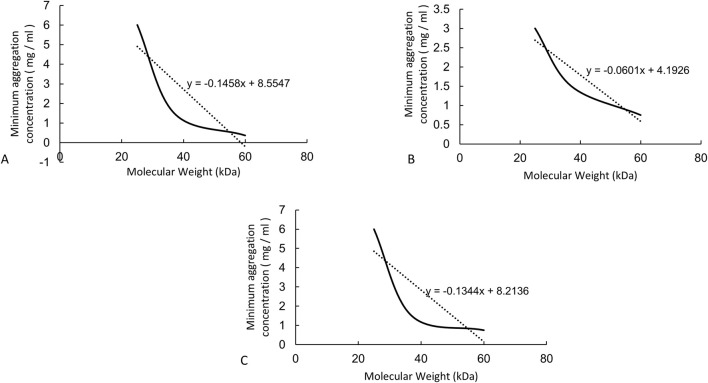
The correlations between molecular weight and concentration required to induce erythrocyte rouleaux formation from animals. Note: **(A)** Curve showing the negative correlation between the lowest concentrations of HA24, HA35, and HA60 that induced human erythrocyte rouleaux formation and molecular weight. The equation of the negative correlation curve is *y* = −0.1458x + 8.5547. **(B)** Curve showing the negative correlation between the lowest concentrations of HA24, HA35, and HA60 that induced beagle erythrocyte rouleaux formation and molecular weight. The equation of the negative correlation curve is *y* = −0.0601 + 4.1926. **(C)** Curve showing the negative correlation between the lowest concentrations of HA24, HA35, and HA60 that induced BALB/c mouse erythrocyte rouleaux formation and molecular weight. The equation of the negative correlation curve is *y* = −0.1344x + 8.2136.

This research collected fresh venous blood from humans, beagles, and BALB/c mice and reacted it with HA24, HA35, and HA60. The three LMW-HA fragments had different minimum concentrations to induce erythrocyte rouleaux formation were different (Table 2). The lowest concentrations of each hyaluronan species required to induce erythrocyte rouleaux formation in different animals over five experiments (n = 6 per experiment) are shown in Figure 2B. When analyzing the scatter plot in Figure 2B alongside the summarized results in Table 2, we observe the following: For humans, the lowest concentrations and corresponding proportions at which HA24, HA35, and HA60 significantly increased erythrocyte rouleaux formation were 6.0 mg/mL (70%), 1.5 mg/mL (66.7%), and 0.375 mg/mL (73.3%), respectively. In beagles, these values were 3.0 mg/mL (66.7%), 1.5 mg/mL (70%), and 0.75 mg/mL (60%), while in BALB/c mice, they were 6.0 mg/mL (53.3%), 1.5 mg/mL (70%), and 0.75 mg/mL (53.3%). Unlike the irregular pattern observed in the lowest concentrations at which HA300 and HA1600 induce erythrocyte aggregation, we identified a negative correlation between the molecular weights of HA24, HA35, and HA60 and the minimum concentration required to induce erythrocyte aggregation by comparing the lowest concentrations at which the highest proportion of erythrocyte rouleaux formation occurred for LMW-HA fragments (Figure 3). In other words, the smaller the molecular weight, the higher the concentration needed to trigger erythrocyte aggregation. This difference is even more pronounced when expressed in terms of molecular concentration. The combined results showed a negative correlation between the molecular weights of HA24, HA35, and HA60 and the lowest concentration at which these LMW-HA fragments induced erythrocyte rouleaux formation in humans, beagles, and BALB/c rats (Figure 3). That is, the smaller the molecular weight, the greater the concentration required to induce erythrocyte aggregation. LMW-HA fragments induced the formation of relatively short erythrocyte rouleaux formation chains (Figures 2Ab) and did not cause erythrocyte rouleaux formation in fresh blood from cattle and sheep (Table 2). The results indicate that the phenomenon of LMW-HA-induced red blood cell aggregation not only exhibits species-specificity, but the aggregation is also not caused by the physical viscosity of LMW-HA.

**TABLE 2:** Minimum concentrations of five hyaluronan species that induced human and difference animals erythrocyte rouleaux formation.

	Final concentration (mg/mL)	Name				
		HA24	HA35	HA60	HA300	HA1600
Human	12.0000	+	+	+	+	+
	6.0000	+	+	+	+	+
	3.0000	—	+	+	—*	+*
	1.5000	—	+	+	+	+
	0.7500	—	—	+	—	—
	0.3750	—	—	+	—	—
	0.1875	—	—	—	—	—
beagle	6.0000	+#	+	+#	+*	+#
	3.0000	+	+	+#	+*	+#
	1.5000	—	+	+	—*	+
	0.7500	—	—	+	—	+
	0.3750	—	—	—	—	+
	0.1875	—	—	—	—	+
BALB/c mouse	6.0000	+	+	+	+	+*
	3.0000	—	+	+	+	+*
	1.5000	—	+	+	+	+*
	0.7500	—	—	+	+	—
	0.3750	—	—	—	—	—
	0.1875	—	—	—	—	—
Bovine	6.0000	—	—	—	+	+
	3.0000	—	—	—	+	+
	1.5000	—	—	—	+	+
	0.7500	—	—	—	—	+
	0.3750	—	—	—	—	—
	0.1875	—	—	—	—	—
Sheep	6.0000	—	—	—	—	+
	3.0000	—	—	—	—	+
	1.5000	—	—	—	—	+
	0.7500	—	—	—	—	+
	0.3750	—	—	—	—	—
	0.1875	—	—	—	—	—

Note: “+” indicates red blood cell curved coin roll; “—” indicates normal cell status; “*” indicates severe cell deformation. “#” indicates large degree of coagulation.

### 3.3 Effect of erythrocyte rouleaux formation blockade

**FIGURE 4:**
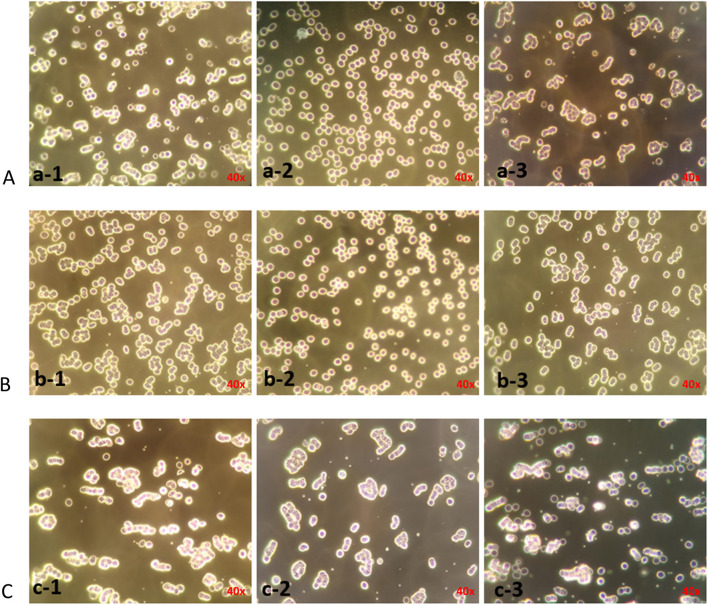
Antibody blockade of erythrocyte rouleaux formation induced by three LMW-HA fragments species. Note: The magnification is ×40. **(A–C)** LMW-HA fragments HA24, HA35, HA60. (a-1), (b-1), (c-1) Erythrocyte agglutination was induced by 6 mg/mL HA24, 1.5 mg/mL HA35, 0.375 mg/mlHA60; (a-2), (b-2), (c-2) 10 μg/mL anti-human CD44 antibody was incubated with erythrocytes for 25 min before the addition of 6 mg/mL HA24, 1.5 mg/mL HA35, 0.375 mg/mlHA60; (a-3), (b-3), (c-3) 10 μg/mL rabbit IgG polyclonal isotype control was incubated with erythrocytes for 25 min before the addition of 6 mg/mL HA24, 1.5 mg/mL HA35, 0.375 mg/mL HA60.

Studies have shown that the CD44 protein is the primary hyaluronan receptor expressed on the surface of erythrocytes. Therefore, the relationship between intercellular forces and HA receptors was indirectly studied at the cellular level. As shown in Figure 4, HA24-, HA35-, and HA60-induced erythrocyte rouleaux formation in human fresh blood (a-1, b-1, c-1) was partially blocked by 10 μg/mL anti-human CD44 antibody (a-2, b-2, c-2). Conversely, erythrocyte rouleaux formation induced by HA24, HA35, and HA60 in human fresh blood (a-1, b-1, c-1) was not inhibited by 10 μg/mL of anti-rabbit CD44 antibody (a-3, b-3, c-3).

**FIGURE 5:**
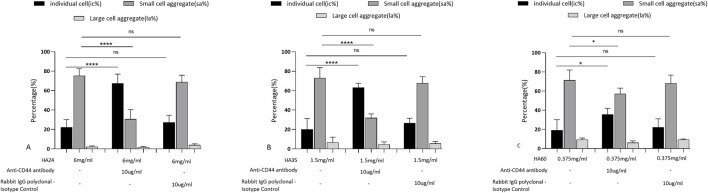
Rates at which antibodies blocked erythrocyte rouleaux formation induced by three LMW-HA formation. Percentage of cells in different states after the addition of 10 μg/mL anti-human CD44 antibody and rabbit IgG polyclonal isotype control compared to HA24 only. **(B)** Antibody blockade rate for HA35-induced erythrocyte rouleaux formation. Percentage of cells in different states after the addition of 10 μg/mL anti-human CD44 antibody and rabbit IgG polyclonal isotype control compared to HA35 only. **(C)** Antibody blockade rate for HA60-induced erythrocyte rouleaux formation. Percentage of cells in different states after the addition of 10 μg/mL anti-human CD44 antibody and rabbit IgG polyclonal isotype control compared to HA60 only. Values are presented as the means ± SDs. Analysis of variance was carried out with Tukey’s test. The increase in ic% is the blockade rate for the single-cell state. Anti-human CD44 antibody: ^****^Comparison between two groups, p < 0.001 (n = 5). The increase in sa% is the blockade rate of the “curved coin roll” state consisting of groups of 8–10 individual cells. Anti-human CD44 antibody: ^****^Comparison between two groups, p < 0.001 (n = 5). The increase in la% is the blockade rate of the “curved coin roll” state consisting of groups of at least 10 single cells.

As shown in Figure 5A, at 6 mg/mL HA24, sa% decreased from 75.4% to 30.6%, and the rouleaux formation rate decreased by 44.8% (P < 0.001). When HA35 was applied at 1.5 mg/mL, sa% decreased from 73% to 32%, and the rouleaux formation rate decreased by 41% (Figure 5B, P < 0.001). HA60 at a concentration of 0.375 mg/mL showed less effective blocking, causing a decrease in sa% from 71.4% to 58.2% and a decline in rouleaux formation rate of only 14.2% (Figure 5C, P < 0.05). The rabbit IgG polyclonal isotype control did not block rouleaux formation (Figures 5A–C, P > 0.05).

### 3.4 The molecular weights of LMW-HA fragments and canine red blood cell sedimentation rate

**FIGURE 6:**
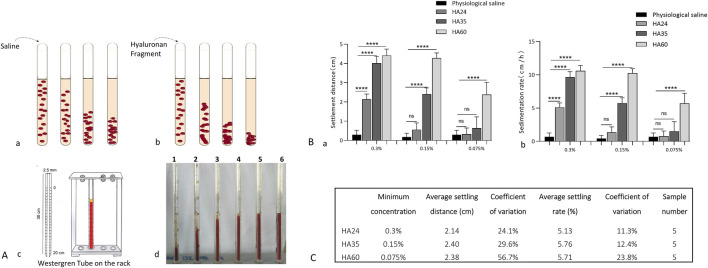
**(A)** Patterns of erythrocyte sedimentation; **(a)** Red blood cell status and sedimentation with the addition of saline. **(b)** The status of erythrocytes when hyaluronan fragments were added and their sedimentation. **(c)** Diagram of the Westergren tube assay. **(d)** Erythrocyte sedimentation distances at 5 min for 0.15% HA24, HA35, HA60, HA300, and HA1600. 1-HA1600, 2-HA300, 3-HA60, 4-HA35, 5-HA24, and 6-saline. **(B)**: Sedimentation distances and sedimentation rates; **(a)** Erythrocyte sedimentation distance induced by 0.3% HA24, 0.15% HA35, and 0.075% HA60. **(b)** ESR induced by 0.3% HA24, 0.15% HA35, and 0.075% HA60. Values are presented as the means ± SDs (n = 5). Sedimentation rate refers to the rate of decline per hour in the stationary state of erythrocytes. **(C)** The minimum concentrations of hyaluronan that induced erythrocyte rouleaux formation at 25 min.

When fresh blood is at rest, red blood cells settle due to gravity (Figure 6A). HA24, HA35, and HA60 were mixed with fresh venous blood from beagles, and incubation at rest not only induced rouleaux formation but also accelerated erythrocyte settling (Figures 6Ab). We found with a Westergren tube assay that the degree to which erythrocyte sedimentation increased varied with the molecular weight of the hyaluronan (Figures 6Ac, d). As shown in Figures 6Ad, C, HA24, HA35, and HA60 induced erythrocyte rouleaux formation to varying degrees at percentage concentrations of 0.3% (3 mg/mL), 0.15% (1.5 mg/mL), and 0.075% (0.75 mg/mL). We used LMW-HA fragments at the lowest concentrations required to induce erythrocyte rouleaux formation as a reference standard. The lowest percentage concentration at which HA24 induced erythrocyte rouleaux formation in dogs was 0.3% (P < 0.05, Figures 6A, B). The average erythrocyte settling distance at 25 min was 2.14 cm, with a coefficient of variation of 24.1% (Figure 6C). The average settling rate was 5.13 mm/h, with a coefficient of variation of 11.3%. The lowest percentage concentration at which HA35 induced erythrocyte rouleaux formation in dogs was 0.15% (P < 0.001, Figures 6Aa, b). The average erythrocyte settling distance at 25 min was 2.40 cm, with a coefficient of variation of 29.6%, and the average settling rate was 5.76 mm/h, with an average coefficient of variation of 12.4%. The lowest percentage concentration at which HA60 induced erythrocyte rouleaux formation in dogs was 0.075% (P < 0.05, Figures 6Aa, b). The mean erythrocyte sedimentation distance at 25 min was 2.38 cm, the mean sedimentation rate was 5.71 mm/h, and the coefficient of variation was 23.8%. The mean sedimentation rates of HA24 and HA35 differed by less than 15%, indicating the reliability of our method. In contrast, the concentration range of HA60 fluctuated more than that of HA24 or HA35 because of its uneven molecular weight distribution.

**FIGURE 7:**
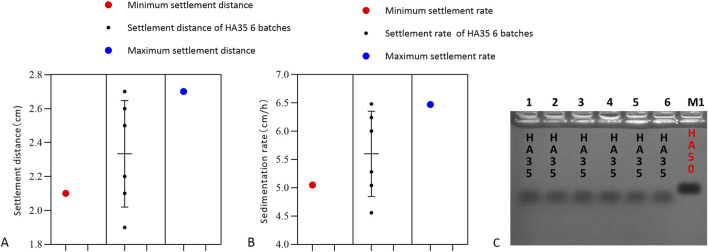
Settling distance, settling rate and molecular weight. **(A)** Sedimentation distance with erythrocyte sedimentation induced by six batches of HA35. The minimum settling distance in the presence of HA35 was 2.1 cm, and the maximum settling distance was 2.7 cm/h (n = 6). **(B)** Sedimentation rate with erythrocyte sedimentation induced by six batches of HA35. The minimum settling rate with HA35 was 5.05 cm/h, and the maximum settling rate was 6.47 cm/h (n = 6). **(C)** Molecular weight of six batches of HA35. M1: 50 kDa molecular weights hyaluronic acid standard (Sigma).

To further validate that the red blood cell sedimentation rate can be used to detect the activity and quality of LMW-HA fragments as raw materials or products, six batches of HA35 injection were mixed with canine red blood cells and diluted to 1.5 mg/mL. The results of the experiment are shown in Figure 7. Among the six batches of HA35 injection, eighty percent gave a settling distance and settling rate within the standard variation for HA35-induced red blood cell sedimentation. Therefore, we could use a final concentration of 1.5 mg/mL HA fragment HA35 to test the quality of the product of HA35 or raw HA35 material. The following quality standards were required for the product of the HA fragment HA35 or the raw HA35 material produced in each batch: an induced red blood cell sedimentation distance between 2.10 and 2.70 cm and a sedimentation rate between 5.05 and 6.47 cm/h at a concentration of 1.5 mg/mL. Therefore, the relationship between the molecular weight and sedimentation rate of LMW-HA fragments could be used to assist in determining not only the molecular weight range of LMW-HA fragments but also the degree of variation in the molecular weights of LMW-HA fragments from different batches. This method was shown to be effective for the quality control of products of the HA fragment HA35 and raw HA35 materials.

## 4 Discussion

The binding strength and interactions of HA with various cell surfaces have highlighted its significance in the medical field (Lesley et al., 2004; Zheng et al., 2023; Dovedytis et al., 2020). Studies have shown that by regulating cell behavior, HA can significantly influence cell migration, and blocking key surface receptors such as CD44, LYVE-1, and RHAMM can inhibit this process (Misra et al., 2015; Chaudhry et al., 2021; Amorim et al., 2018; Merino-Casallo et al., 2022). HA has also demonstrated strong biological activity (Ye et al., 2024). For example, in regular mice genetically modified with the Has2 gene from the naked mole rat, the accumulation of HMW-HA significantly extended their lifespan (Zhang et al., 2023). However, while much research focuses on HMW-HA, low molecular weight HA35, derived from human colostrum (Hill et al., 2012; Hill et al., 2013; Bellar et al., 2019), has shown remarkable analgesic effects due to its excellent tissue permeability, high purity, and endotoxin free properties (Xu et al., 2024; Treger et al., 2024; Zhang et al., 2024; Dashnyam et al., 2023). This enhanced biological activity is likely due to HA35’s ability to promote neutrophil and lymphocyte migration, which reduces inflammation, redness, and pain (Gantumur et al., 2024). Additionally, HA35 alleviates pain by binding to the capsaicin receptor TRPV1 ion channel and inhibiting calcium (Ca2+) influx (Caires et al., 2015; de la Peña et al., 2016). Thus, HA’s biological activities are closely related to its binding strength and interactions with cell surfaces.

This capacity extends to red blood cell physiology, where HA’s interactions with erythrocytes unveil unique aspects of blood rheology and cell aggregation behaviors. Building on these findings, this study further investigates how different molecular weights of HA interact with red blood cells, revealing that HA can induce erythrocyte rouleaux formation (Jia et al., 2023; Baskurt and Meiselman, 2013). This phenomenon typically occurs under conditions that increase blood viscosity, such as low temperatures, hypoxia, or elevated concentrations of plasma proteins like fibrinogen and globulin, causing red blood cells to aggregate (Nader et al., 2019). Recent reports have documented an increased incidence of stroke and vascular occlusion associated with HA aggregation, particularly in aesthetic medicine applications such as dermal fillers. Cases of HA inadvertently entering the vascular system have led to severe complications, including retinal artery occlusion and ischemic stroke (Grzybinski and Temin, 2018; Turcza et al., 2025). Physiological aggregation is usually reversible, with red blood cells dispersing once conditions normalize. However, in pathological conditions such as inflammation, infection, or autoimmune diseases, red blood cell aggregation can become irreversible (Nader et al., 2019). This study indicates that LMW-HA-induced erythrocyte aggregation is reversible, the minimum effective concentration for erythrocyte aggregation increases as molecular weight decreases, and it can be partially blocked by exogenous CD44 antibodies (Kerfoot et al., 2008). Steven’s study suggests that the altered adhesion or aggregation tendency of red blood cells may be due to LMW-HA binding to CD44, which subsequently modifies the properties of the erythrocyte membrane and induces conformational changes (Kerfoot et al., 2008). CD44 is a primary HA receptor known to mediate cellular responses through structural alterations upon ligand binding (Misra et al., 2015; Chaudhry et al., 2021; Amorim et al., 2018). Other potential erythrocyte receptors, such as ICAM-4 (Ihanus et al., 2007) and integrin α4β1 (Durpès et al., 2011), may regulate erythrocyte aggregation through cooperative interactions with CD44. However, its binding affinity to HA requires further investigation using molecular docking and surface plasmon resonance (SPR) analysis, as well as validation through flow cytometry and co-immunoprecipitation assays. Preliminary CD44-blocking experiments indicate that HA interacts with red blood cells by binding to their surface and that different LMW-HA fragments exhibit varying binding capacities. Notably, the minimum concentration required for different LMW-HA fragments to induce red blood cell aggregation also varies. Unlike the irregular aggregation patterns seen with medium to HMW-HA in different animal species, LMW-HA shows more consistency in binding. Across different species (human, beagle dog, and mouse), HA60 consistently exhibited the strongest binding capacity, HA24 the weakest, and HA35 was intermediate.

The study found that LMW-HA did not exhibit similar binding or aggregation effects on bovine and ovine red blood cells, confirming the species specificity of LMW-HA (Guo et al., 2022) and suggesting that red blood cell aggregation is not driven by HA’s adhesive properties (Kerfoot et al., 2008). Unpublished clinical studies on HA35 have demonstrated its effectiveness in treating human intestinal obstruction, yet previous studies on HA35 in treating intestinal obstruction in 16 Beagle dogs showed minimal success (Wang et al., 2022). This highlights the potential species-specific nature of HA’s binding to cell surfaces or receptors, as well as its signaling and biological effects (Cowman et al., 2015; Kobayashi et al., 2020; Fraser et al., 1997). These findings suggest that the biological effects of LMW-HA may vary between humans and animals, or even among different animal species. In other words, while LMW-HA with good tissue permeability may hold therapeutic potential, its effects may differ across species, warranting further clinical research to confirm its efficacy. The inclusion of multiple species in this study was critical to assess the consistency and potential variability of LMW-HA interactions, as differences in erythrocyte surface receptors and plasma composition among species may influence aggregation behavior. These cross-species experiments not only highlight the translational relevance of our findings but also underscore the need to further investigate potential off-target effects that may arise due to interspecies differences.

Although LMW-HA promotes red blood cell aggregation, it does not trigger coagulation. This implies that, at certain concentrations, LMW-HA could offer a safer alternative to HMW-HA injections, with a lower risk of clotting-related complications. Additionally, animal experiments revealed that within 5 min of subcutaneous injection, 125I-labeled HA35 could be detected in the spleen and lymph nodes of mice (Gantumur et al., 2024), demonstrating excellent tissue permeability. Furthermore, HA24, HA35, and HA60 all passed through a 220 nm filter membrane without resistance (Gantumur et al., 2024), while medium-molecular-weight HA and HMW-HA could not pass through, even at the lowest concentrations. This further highlights the superior tissue permeability of LMW-HA. As a result, LMW-HA exhibits significantly better drugforming potential *in vitro* compared to HMW-HA. However, while this study preliminarily demonstrated that different molecular weights of LMW-HA have varying binding activities, further clinical studies, molecular imaging, TRPV1 calcium channel assays, and lymphatic transport tests are necessary to fully validate the specific biological activity and pharmaceutical potential of these molecules. This research offers a valuable foundation for determining the optimal molecular weight of LMW-HA for drug development.

This study also contributes an important reference for detecting and controlling the molecular weight range of LMW-HA. It is well known that methods for detecting HA include intrinsic viscosity, GPC, agarose gel electrophoresis, and GPC-MALLS (Wang et al., 2019). The intrinsic viscosity method, as outlined in the European Pharmacopoeia, is effective for measuring HMW-HA (Brûlé et al., 2021), but its accuracy is limited when measuring LMW-HA. In this study, we utilized gel electrophoresis and GPC-MALLS to determine the molecular weights of three LMW-HA types, revealing that red blood cell aggregation was related to both the molecular weight and the binding strength of the HA to cell surfaces. Specifically, the minimum concentration of LMW-HA required to induce red blood cell aggregation in different animals was negatively correlated with its molecular weight. Using the minimum concentration needed to induce aggregation in one animal’s red blood cells as a reference, we found that the variation in average sedimentation rate for HA24 at 3 mg/mL and HA35 at 0.15 mg/mL was less than 15%. This suggests that HA24 and HA35 obtained through enzymatic methods have high molecular weight purity, whereas HA60 produced through physical methods has lower purity (Valcarcel et al., 2020; Gottschalk et al., 2022). Comparing six batches of specific molecular weight products, we found that the minimum concentration to induce canine red blood cell aggregation, as well as the sedimentation distance and rate, remained within the standard variation range (Figure 7). Therefore, this study establishes a highly sensitive quality control method for monitoring the activity and molecular weight consistency of LMW-HA raw materials or products across different batches.

In conclusion, the results of this study suggest that the experimental methods used to assess the binding of differentsized LMW-HA to red blood cell surfaces could serve as an effective approach for evaluating their biological activity. However, in clinical applications, intravenous administration should be avoided to prevent rapid absorption or excessively high local concentrations, which may induce red blood cell aggregation. Subcutaneous or localized injections may represent safer and more effective methods of delivering LMW-HA (Xu et al., 2024; Treger et al., 2024). These findings provide a foundation for the further development of HA35 and HA60 as potential injectable or oral therapeutics.

## Data Availability

The original contributions presented in the study are included in the article/supplementary material, further inquiries can be directed to the corresponding author.

## References

[B1] Amorim S. da Costa D. S. Freitas D. Reis C. A. Reis R. L. Pashkuleva I. (2018). Molecular weight of surface-immobilized hyaluronic acid influences CD44-mediated binding of gastric cancer cells. Sci. Rep. 8 (1), 16058. 10.1038/s41598-018-34445-0 30375477 PMC6207784

[B2] Barshtein G. Zelig O. Gural A. Arbell D. Yedgar S. (2022). Determination of red blood cell adhesion to vascular endothelial cells: a critical role for blood plasma. Colloids Surf. B Biointerfaces 210, 112226. 10.1016/j.colsurfb.2021.112226 34836705

[B3] Baskurt O. K. Meiselman H. J. (2013). Erythrocyte aggregation: basic aspects and clinical importance. Clin. Hemorheol. Microcirc. 53 (1-2), 23–37. 10.3233/CH-2012-1573 22975932

[B4] Bellar A. Kessler S. P. Obery D. R. Sangwan N. Welch N. Nagy L. E. (2019). Safety of Hyaluronan 35 in healthy human subjects: a pilot study. Nutrients 11, 1135. 10.3390/nu11051135 31121841 PMC6566413

[B5] Brûlé S. Leroux R. England P. Raynal B. (2021). Protein intrinsic viscosity determination with the viscosizer TD instrument: reaching beyond the initially expected applications. Eur. Biophys. J. 50 (3-4), 587–595. 10.1007/s00249-020-01492-3 33486532

[B6] Caires R. Luis E. Taberner F. J. Fernandez-Ballester G. Ferrer-Montiel A. Balazs E. A. (2015). Hyaluronan modulates TRPV1 channel opening, reducing peripheral nociceptor activity and pain. Nat. Commun. 6, 8095. 10.1038/ncomms9095 26311398 PMC4560824

[B7] Campo G. M. Avenoso A. D'Ascola A. Scuruchi M. Prestipino V. Nastasi G. (2012). The inhibition of hyaluronan degradation reduced pro-inflammatory cytokines in mouse synovial fibroblasts subjected to collagen-induced arthritis. J. Cell Biochem. 113 (6), 1852–1867. 10.1002/jcb.24054 22234777

[B8] Chaudhry G. E. Akim A. Zafar M. N. Safdar N. Sung Y. Y. Muhammad T. S. T. (2021). Understanding hyaluronan receptor (CD44) interaction, HA-CD44 activated potential targets in cancer therapeutics. Adv. Pharm. Bull. 11 (3), 426–438. 10.34172/apb.2021.050 34513617 PMC8421618

[B9] Cowman M. K. Lee H. G. Schwertfeger K. L. McCarthy J. B. Turley E. A. (2015). The content and size of hyaluronan in biological fluids and tissues. Front. Immunol. 6, 261. 10.3389/fimmu.2015.00261 26082778 PMC4451640

[B10] Cyphert J. M. Trempus C. S. Garantziotis S. (2015). Size matters: molecular weight specificity of hyaluronan effects in cell biology. Int. J. Cell Biol. 2015 (7), 563818–8. 10.1155/2015/563818 26448754 PMC4581549

[B11] Dashnyam K. Treger D. Jia X. X. Gantumur M. A. Bayanmunkh A. Hui J. (2023). Injection of 35kDa hyaluronan fragment alleviates pain associated with radiotherapy for treatment of colorectal and rectal cancer. Pharmacol. Pharmacogenomics 1 (1), 131–135. 10.31488/JPP.106

[B12] de la Peña E. Gomis A. Ferrer-Montiel A. Belmonte C. (2016). TRPV1 channel modulation by hyaluronan reduces pain. Channels (Austin) 10 (2), 81–82. 10.1080/19336950.2015.1109300 26517313 PMC4960985

[B13] Diamanti D. Pieroni C. Pennisi M. G. Marchetti V. Gori E. Paltrinieri S. (2025). The erythrocyte sedimentation rate (ESR) in veterinary medicine: a focused review in dogs and cats. Anim. (Basel) 15 (2), 246. 10.3390/ani15020246 PMC11758338 39858246

[B14] Dovedytis M. Liu Z. Bartlett S. (2020). Hyaluronic acid and its biomedical applications: a review. Eng. Regen. 1 (12), 102–113. 10.1016/j.engreg.2020.10.001

[B15] Durpès M. C. Hardy-Dessources M. D. El N. W. Picot J. Lemonne N. Elion J. (2011). Activation state of alpha4beta1 integrin on sickle red blood cells is linked to the duffy antigen receptor for chemokines (DARC) expression. J. Biol. Chem. 286 (4), 3057–3064. 10.1074/jbc.M110.173229 21088296 PMC3024799

[B16] Flormann D. Aouane O. Kaestner L. Ruloff C. Misbah C. Podgorski T. (2017). The buckling instability of aggregating red blood cells. Sci. Rep. 7 (1), 7928. 10.1038/s41598-017-07634-6 28801570 PMC5554189

[B17] Fraser J. R. Laurent T. C. Laurent U. B. (1997). Hyaluronan: its nature, distribution, functions, and turnover. J. Intern. Med. 242, 27–33. 10.1046/j.1365-2796.1997.00170.x 9260563

[B18] Gantumur M. A. Jia X. Hui J. H. Barber C. Wan L. Furenlid L. R. (2024). Characterization, bioactivity, and biodistribution of 35 kDa hyaluronan fragment. Life (Basel) 14 (1), 97. 10.3390/life14010097 38255712 PMC10817694

[B19] Garantziotis S. Savani R. C. (2019). Hyaluronan biology: a complex balancing act of structure, function, location and context. Matrix Biol. 78–79, 1–10. 10.1016/j.matbio.2019.02.002 PMC6774756 30802498

[B20] Ghosh S. Samarasinghe A. E. Hoselton S. A. Dorsam G. P. Schuh J. M. (2014). Hyaluronan deposition and co-localization with inflammatory cells and collagen in a murine model of fungal allergic asthma. Inflamm. Res. 63 (6), 475–484. 10.1007/s00011-014-0719-3 24519432 PMC4020973

[B21] Gillespie A. H. Doctor A. (2021). Red blood cell contribution to hemostasis. Front. Pediatr. 9, 629824. 10.3389/fped.2021.629824 33869111 PMC8047051

[B22] Gottschalk J. Aßmann M. Kuballa J. Elling L. (2022). Repetitive synthesis of high-molecular-weight hyaluronic acid with immobilized enzyme cascades. ChemSusChem 15 (9), e202101071. 10.1002/cssc.202101071 34143936 PMC9290584

[B23] Grzybinski S. Temin E. (2018). Vascular occlusion after hyaluronic acid filler injection. Clin. Pract. Cases Emerg. Med. 2 (2), 167–168. 10.5811/cpcem.2018.2.37149 29849280 PMC5965121

[B24] Guo T. T. Wang J. Q. Jia X. X. (2022). Inducing effect of low-molecular-weight hyaluronic acid fragments on red blood cell aggregation and its species specificity. J. Qingdao Agric. Univ. (Nat. Sci. Ed.) 39 (2), 96–108. 10.3969/J.ISSN.1674-148X.2022.02.004

[B25] Hasiwa N. Bailey J. Clausing P. Daneshian M. Eileraas M. Farkas S. (2011). Critical evaluation of the use of dogs in biomedical research and testing in Europe. ALTEX 28 (4), 326–340. 10.14573/altex.2011.4.326 22130483

[B26] Hill D. R. Kessler S. P. Rho H. K. Cowman M. K. de la Motte C. A. (2012). Specific-sized hyaluronan fragments promote expression of human β-defensin 2 in intestinal epithelium. J. Biol. Chem. 287, 30610–30624. 10.1074/jbc.M112.356238 22761444 PMC3436307

[B27] Hill D. R. Rho H. K. Kessler S. P. Amin R. Homer C. R. McDonald C. (2013). Human milk hyaluronan enhances innate defense of the intestinal epithelium. J. Biol. Chem. 288, 29090–29104. 10.1074/jbc.M113.468629 23950179 PMC3790008

[B28] Iaconisi G. N. Lunetti P. Gallo N. Cappello A. R. Fiermonte G. Dolce V. (2023). Hyaluronic acid: a powerful biomolecule with wide-ranging applications-A comprehensive review. Int. J. Mol. Sci. 24 (12), 10296. 10.3390/ijms241210296 37373443 PMC10299688

[B29] Ihanus E. Uotila L. M. Toivanen A. Varis M. Gahmberg C. G. (2007). Red-cell ICAM-4 is a ligand for the monocyte/macrophage integrin CD11c/CD18: characterization of the binding sites on ICAM-4. Blood 109 (2), 802–810. 10.1182/blood-2006-04-014878 16985175

[B30] Jia X. Shi M. Wang Q. Hui J. Shofaro J. H. Erkhembayar R. (2023). Anti-inflammatory effects of the 35kDa hyaluronic acid fragment (B-HA/HA35). J. Inflamm. Res. 16, 209–224. 10.2147/JIR.S393495 36686276 PMC9846287

[B31] Johnson P. Arif A. A. Lee-Sayer S. S. M. Dong Y. (2018). Hyaluronan and its interactions with immune cells in the healthy and inflamed lung. Front. Immunol. 9, 2787. 10.3389/fimmu.2018.02787 30555472 PMC6281886

[B32] Kerfoot S. M. McRae K. Lam F. McAvoy E. F. Clark S. Brain M. (2008). A novel mechanism of erythrocyte capture from circulation in humans. Exp. Hematol. 36 (2), 111–118. 10.1016/j.exphem.2007.08.029 18037227

[B33] Klei R. L. T. Dalimot J. J. Beuger B. M. Veldthuis M. Ichou F. A. Verkuijlen P. J. J. H. (2020). The Gardos effect drives erythrocyte senescence and leads to Lu/BCAM and CD44 adhesion molecule activation. Blood Adv. 4 (24), 6218–6229. 10.1182/bloodadvances.2020003077 33351118 PMC7757008

[B34] Kobayashi T. Chanmee T. Itano N. (2020). Hyaluronan: metabolism and function. Biomolecules 10 (11), 1525. 10.3390/biom10111525 33171800 PMC7695009

[B35] Kratz A. Plebani M. Peng M. Lee Y. K. McCafferty R. Machin S. J. (2017). ICSH recommendations for modified and alternate methods measuring the erythrocyte sedimentation rate. Int. J. Lab. Hematol. 39 (5), 448–457. 10.1111/ijlh.12693 28497537

[B36] Kulaberoglu Y. Bhushan B. Hadi F. Chakrabarti S. Khaled W. T. Rankin K. S. (2019). The material properties of naked mole-rat hyaluronan. Sci. Rep. 9 (1), 6632. 10.1038/s41598-019-43194-7 31036852 PMC6488695

[B37] Lazari D. Freitas Leal J. K. Brock R. Bosman G. (2020). The relationship between aggregation and deformability of red blood cells in health and disease. Front. Physiol. 11, 288. 10.3389/fphys.2020.00288 32351399 PMC7174766

[B38] Lesley J. Gál I. Mahoney D. J. Cordell M. R. Rugg M. S. Hyman R. (2004). TSG-6 modulates the interaction between hyaluronan and cell surface CD44. J. Biol. Chem. 279 (24), 25745–25754. 10.1074/jbc.M313319200 15060082

[B39] Li L. Zhao B. Feng Z. Wang D. Yuan T. Song G. (2025). Role and influence mechanism of different concentration of hyaluronic acid on physicochemical and organoleptic properties of yogurt. J. Dairy Sci. 108 (1), 218–228. 10.3168/jds.2024-25687 39414018

[B40] Marcellin E. Steen J. A. Nielsen L. K. (2014). Insight into hyaluronic acid molecular weight control. Appl. Microbiol. Biotechnol. 98 (16), 6947–6956. 10.1007/s00253-014-5853-x 24957250

[B41] Merino-Casallo F. Gomez-Benito M. J. Hervas-Raluy S. Garcia-Aznar J. M. (2022). Unravelling cell migration: defining movement from the cell surface. Cell Adh. Migr. 16 (1), 25–64. 10.1080/19336918.2022.2055520 35499121 PMC9067518

[B42] Misra S. Hascall V. C. Markwald R. R. Ghatak S. (2015). Interactions between hyaluronan and its receptors (CD44, RHAMM) regulate the activities of inflammation and cancer. Front. Immunol. 6, 201. 10.3389/fimmu.2015.00201 25999946 PMC4422082

[B43] Nader E. Skinner S. Romana M. Fort R. Lemonne N. Guillot N. (2019). Blood rheology: key parameters, impact on blood flow, role in sickle cell disease, and effects of exercise. Front. Physiol. 10, 1329. 10.3389/fphys.2019.01329 31749708 PMC6842957

[B44] Ruby J. G. Smith M. Buffenstein R. (2018). Naked mole-rat mortality rates defy gompertzian laws by not increasing with age. Elife 7, e31157. 10.7554/eLife.31157 29364116 PMC5783610

[B45] Seluanov A. Gladyshev V. N. Vijg J. Gorbunova V. (2018). Mechanisms of cancer resistance in long-lived mammals. Nat. Rev. Cancer 18 (7), 433–441. 10.1038/s41568-018-0004-9 29622806 PMC6015544

[B46] Shen M. Q. Liu X. Wei C. (2015). Clinical study of 35kDa hyaluronan fragment B-HA on laser-induced inflammatory skin wound. Prog. Mod. Bio. 15 (7), 1300–1304.

[B47] Smith E. S. Omerbašić D. Lechner S. G. Anirudhan G. Lapatsina L. Lewin G. R. (2011). The molecular basis of acid insensitivity in the African naked mole-rat. Science 334 (6062), 1557–1560. 10.1126/science.1213760 22174253

[B48] Solis M. A. Chen Y. H. Wong T. Y. Bittencourt V. Z. Lin Y. C. Huang L. L. (2012). Hyaluronan regulates cell behavior: a potential niche matrix for stem cells. Biochem. Res. Int. 2012, 346972. 10.1155/2012/346972 22400115 PMC3287012

[B49] Takasugi M. Firsanov D. Tombline G. Ning H. Ablaeva J. Seluanov A. (2020). Naked mole-rat very-high-molecular-mass hyaluronan exhibits superior cytoprotective properties. Nat. Commun. 11 (1), 2376. 10.1038/s41467-020-16050-w 32398747 PMC7217962

[B50] Tian X. Azpurua J. Hine C. Vaidya A. Myakishev-Rempel M. Ablaeva J. (2013). High-molecular-mass hyaluronan mediates the cancer resistance of the naked mole rat. Nature 499 (7458), 346–349. 10.1038/nature12234 23783513 PMC3720720

[B51] Treger D. Zhang L. Jia X. Hui J. H. Gantumur M. A. Hui M. (2024). A clinical study of the local injection of a freshly manufactured 35 kDa hyaluronan fragment for treating chronic wounds. Int. Wound J. 21 (5), e14906. 10.1111/iwj.14906 38745342 PMC11093919

[B52] Turcza J. F. Bartosinska J. Raczkiewicz D. (2025). Critical ischemia following hyaluronic acid filler injection: a case report. J. Clin. Med. 14, 802. 10.3390/jcm14030802 39941473 PMC11818802

[B53] Valcarcel J. García M. R. Varela U. R. Vázquez J. A. (2020). Hyaluronic acid of tailored molecular weight by enzymatic and acid depolymerization. Int. J. Biol. Macromol. 145, 788–794. 10.1016/j.ijbiomac.2019.12.221 31887382

[B54] Wang F. Wang F. W. Cui J. Y. (2019). Comparative study on four molecular weight detection methods of small molecular weight hyaluronic acid fragments. Strait Pharm. J. 31, 89–93.

[B55] Wang J. Q. Guo T. T. Hui X. H. (2022). The effect of 35 kDa hyaluronic acid fragments on lipopolysaccharide-induced inflammation in dogs. J. Qingdao Agric. Univ. (Nat. Sci. Ed.) 39 (2), 109–113. 10.3969/J.ISSN.1674-148X.2022.02.005

[B56] Xu F. H. Treger D. Ma X. Y. Jia X. X. Shofaro J. H. Ganbaatar T. (2024). Local injection of a freshly manufactured 35 kDa hyaluronan fragment reduces neuropathic and inflammatory pain: a clinical study. Eur. J. Inflamm. 22. 10.1177/1721727x241258268

[B57] Xu H. Q. Salazar D. M. M. Xu C. X. (2022). Investigation of cell concentration change and cell aggregation due to cell sedimentation during inkjet-based bioprinting of cell-laden bioink. Machines 10 (5), 315. 10.3390/machines10050315

[B58] Ye H. Zhang R. Zhang C. Xia Y. Jin L. (2024). Advances in hyaluronic acid: bioactivity, complexed biomaterials and biological application: a review. Asian J. Surg. S1015–9584 (24), 01841–01844. 10.1016/j.asjsur.2024.10.011 39217010

[B59] Zhang H. Zhang N. Dai Z. Wang Z. Zhang X. Liang X. (2022). Hyaluronic acids mediate the infiltration, migration, and M2 polarization of macrophages: evaluating metabolic molecular phenotypes in gliomas. Mol. Oncol. 16 (22), 3927–3948. 10.1002/1878-0261.13315 36134697 PMC9718117

[B60] Zhang Z. Jia X. Treger D. Hui M. (2024). Low molecular weight 35 kDa hyaluronan fragment HA35 in the treatment of bone metastasis pain: a case report. Med. Baltim. 103 (31), e39145. 10.1097/MD.0000000000039145 PMC11296423 39093812

[B61] Zhang Z. Tian X. Lu J. Y. Boit K. Ablaeva J. Zakusilo F. T. (2023). Increased hyaluronan by naked mole-rat Has2 improves healthspan in mice. Nature 621 (7977), 196–205. 10.1038/s41586-023-06463-0 37612507 PMC10666664

[B62] Zheng X. Wang B. Tang X. Mao B. Zhang Q. Zhang T. (2023). Absorption, metabolism, and functions of hyaluronic acid and its therapeutic prospects in combination with microorganisms: a review. Carbohydr. Polym. 299, 120153. 10.1016/j.carbpol.2022.120153 36876779

